# Author Correction: Deep-learning electronic-structure calculation of magnetic superstructures

**DOI:** 10.1038/s43588-023-00472-9

**Published:** 2023-06-01

**Authors:** He Li, Zechen Tang, Xiaoxun Gong, Nianlong Zou, Wenhui Duan, Yong Xu

**Affiliations:** 1 State Key Laboratory of Low Dimensional Quantum Physics and Department of Physics, Tsinghua University, Beijing, China; 2 Tencent Quantum Laboratory, Tencent, Shenzhen, China; 3 Institute for Advanced Study, Tsinghua University, Beijing, China; 4 School of Physics, Peking University, Beijing, China; 5 Frontier Science Center for Quantum Information, Beijing, China; 6 RIKEN Center for Emergent Matter Science (CEMS), Wako, Japan

**Keywords:** Electronic properties and materials, Electronic structure, Computational methods

Correction to: *Nature Computational Science* 10.1038/s43588-023-00424-3. Published online 26 April 2023.

This paper was originally published under a standard Springer Nature license (© The Author(s), under exclusive licence to Springer Nature America, Inc.). It is now available as an open-access paper under a Creative Commons Attribution 4.0 International license, © The Author(s). The error has been corrected in the HTML and PDF versions of the article.

