# Supplementary material for: Deep-learning electronic-structure calculation of magnetic superstructures
Source: Nat Comput Sci. 2023 Apr 26;3(4):321–7. doi: 10.1038/s43588-023-00424-3 (PMC10766521; doi:10.1038/s43588-023-00424-3)
Supplement: Supplementary file 1 — Details of computational methods and results, including Supplementary Sections 1–9, Figs. 1–13 and Tables 1–3. [file 43588_2023_424_MOESM1_ESM.pdf]

nature computational science

Brief Communication https://doi.org/10.1038/s43588-023-00424-3

Deep-learning electronic-structure calculation of magnetic superstructures

In the format provided by the authors and unedited

This PDF file includes:
Supplementary Sections 1 to 9
Supplementary Figures 1 to 13
Supplementary Tables 1 to 3

Contents

Supplementary Section 1. Nearsightedness nature of magnetism 2

Supplementary Section 2. Time-reversal equivariance 2
Supplementary Section 2.1. Equivariant requirement 2
Supplementary Section 2.2. Complex conjugate of representation matrix 3
Supplementary Section 2.3. Change of basis 3
Supplementary Section 2.4. Realization of equivariance 4

Supplementary Section 3. Ablation studies on the magnetic moment layer with strict locality 4

Supplementary Section 4. Results of example studies 5
Supplementary Section 4.1. Monolayer $NiBr_2$ 5
Supplementary Section 4.2. Monolayer CrI_3 6
Supplementary Section 4.3. CrI_3 nanotubes 7
Supplementary Section 4.4. Moiré-twisted bilayer CrI_3 8

Supplementary Section 5. Computational cost comparison between xDeepH and DFT 9

Supplementary Section 6. Basis set tests 9
Supplementary Section 6.1. Convergence tests of basis set 9
Supplementary Section 6.2. Training efficiency versus the size of basis set 10

Supplementary Section 7. Comparison between xDeepH and SpookyNet 10

Supplementary Section 8. Summary of the dataset 11

Supplementary Section 9. Discussion on applications of xDeepH 11
Supplementary Section 9.1. Study of magnetic alloys 11
Supplementary Section 9.2. Study of many-body interactions 12
Supplementary Section 9.3. Study of collinear magnetic systems 12
Supplementary Section 9.4. Study of non-periodic systems 13

References 13

Supplementary Section 1. Nearsightedness nature of magnetism

We performed numerical tests to check the nearsightedness length of the influence of changing magnetic moment orientation. Density functional theory (DFT) Hamiltonian matrix elements of two analogous magnetic configurations of CrI_3 were compared. The two magnetic configurations are identical, except that the magnetic moment of one Cr atom is reversed. Supplementary Figure 1 displays averaged changes of Hamiltonian matrix elements $[H_{ij}]^{p_1p_2;l_1l_2}_{m_1\sigma_1,m_2\sigma_2}$ with respect to distance. Here, the distance is averaged between r_{ik} and r_{jk}, where the atoms i and j are involved in H_{ij} and the atom k denotes the specified magnetic site with reversed magnetic moment. Changes of Hamiltonian matrix elements are grouped by intervals of 1 Å and averaged over the absolute change. The changes decay to sub-meV within several Å.

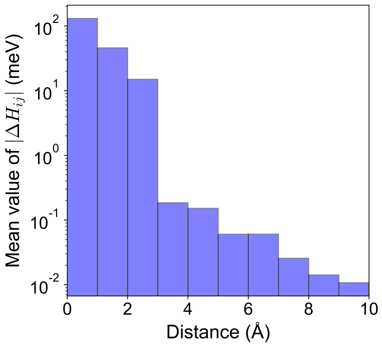

Supplementary Figure 1. The nearsightedness length of the influence of changing magnetic moment orientation. Averaged change of Hamiltonian matrix elements ($|\Delta H_{ij}|$) induced by reversing a local magnetic moment, which decays quickly with increasing distance.

Supplementary Section 2. Time-reversal equivariance

Supplementary Section 2.1. Equivariant requirement

Since real-valued localized atomic orbitals are used in this work, the time-reversal operation works only on the spin degrees of freedom of Hamiltonian martix sub-blocks. The Hamiltonian matrix sub-block $\mathbf{h}^{l_1l_2}_{m_1\sigma_1,m_2\sigma_2}$ can be viewed as a 2×2 matrix $\mathbf{h} = \begin{pmatrix} \mathbf{h}_{\uparrow\uparrow} & \mathbf{h}_{\uparrow\downarrow} \\ \mathbf{h}_{\downarrow\uparrow} & \mathbf{h}_{\downarrow\downarrow} \end{pmatrix}$, where $\mathbf{h}_{\uparrow\uparrow}, \mathbf{h}_{\uparrow\downarrow}, \mathbf{h}_{\downarrow\uparrow}, \mathbf{h}_{\downarrow\downarrow}$ are $(2l_1+1)\times(2l_2+1)$ matrices. The time-reversal operator is $\mathrm{i}\sigma_y K = \begin{pmatrix} 0 & 1 \\ -1 & 0 \end{pmatrix} K$, where K denotes complex conjugation. Under time-reversal, the Hamiltonian matrix sub-block will transform:

$$\begin{pmatrix} \mathbf{h}_{\uparrow\uparrow} & \mathbf{h}_{\uparrow\downarrow} \\ \mathbf{h}_{\downarrow\uparrow} & \mathbf{h}_{\downarrow\downarrow} \end{pmatrix} \xrightarrow{\mathcal{T}} (\mathrm{i}\sigma_y)\, K \begin{pmatrix} \mathbf{h}_{\uparrow\uparrow} & \mathbf{h}_{\uparrow\downarrow} \\ \mathbf{h}_{\downarrow\uparrow} & \mathbf{h}_{\downarrow\downarrow} \end{pmatrix} K\, (\mathrm{i}\sigma_y)^\dagger = \begin{pmatrix} \mathbf{h}^*_{\downarrow\downarrow} & -\mathbf{h}^*_{\downarrow\uparrow} \\ -\mathbf{h}^*_{\uparrow\downarrow} & \mathbf{h}^*_{\uparrow\uparrow} \end{pmatrix}, \tag{1}$$

where "$*$" denotes the complex conjugate.

The 2×2 matrix $\mathbf{h}$ can be viewed as a vector $\mathbf{v}^{\frac{1}{2}\otimes\frac{1}{2}}$ under the basis set of $\{|\uparrow\uparrow\rangle, |\uparrow\downarrow\rangle, |\downarrow\uparrow\rangle, |\downarrow\downarrow\rangle\}$, giving $\mathbf{v}^{\frac{1}{2}\otimes\frac{1}{2}} = \begin{pmatrix} \mathbf{h}_{\uparrow\uparrow} \\ \mathbf{h}_{\uparrow\downarrow} \\ \mathbf{h}_{\downarrow\uparrow} \\ \mathbf{h}_{\downarrow\downarrow} \end{pmatrix}$. The equivariant transformation of time reversal becomes

$$\mathbf{v}^{\frac{1}{2}\otimes\frac{1}{2}} = \begin{pmatrix} \mathbf{h}_{\uparrow\uparrow} \\ \mathbf{h}_{\uparrow\downarrow} \\ \mathbf{h}_{\downarrow\uparrow} \\ \mathbf{h}_{\downarrow\downarrow} \end{pmatrix} \xrightarrow{\mathcal{T}} \begin{pmatrix} 0 & 0 & 0 & 1 \\ 0 & 0 & -1 & 0 \\ 0 & -1 & 0 & 0 \\ 1 & 0 & 0 & 0 \end{pmatrix} K\mathbf{v}^{\frac{1}{2}\otimes\frac{1}{2}} = \begin{pmatrix} \mathbf{h}^*_{\downarrow\downarrow} \\ -\mathbf{h}^*_{\downarrow\uparrow} \\ -\mathbf{h}^*_{\uparrow\downarrow} \\ \mathbf{h}^*_{\uparrow\uparrow} \end{pmatrix} \tag{2}$$

Supplementary Section 2.2. Complex conjugate of representation matrix

We expect to construct Hamiltonian matrix sub-blocks whose spin degrees of freedom are transformed in this way under rotation $\mathbf{R}$:

$$\mathbf{h}_{\sigma_1\sigma_2} \xrightarrow{\mathbf{R}} D^{\frac{1}{2}}_{\sigma_1\sigma_1'}(\mathbf{R}) \left[D^{\frac{1}{2}}_{\sigma_2\sigma_2'}(\mathbf{R})\right]^* \mathbf{h}'_{\sigma_1\sigma_2}, \tag{3}$$

where D is the Wigner-D matrix and the prime on the matrix $\mathbf{h}_{\sigma_1\sigma_2}$ means that its orbital degrees of freedom have been rotated. However, the equivariant matrix $\tilde{\mathbf{h}}_{\sigma_1\sigma_2}$ constructed by the tensor product is transformed like:

$$\tilde{\mathbf{h}}_{\sigma_1\sigma_2} \xrightarrow{\mathbf{R}} \sum_{\sigma_1'\sigma_2'} D^{\frac{1}{2}}_{\sigma_1\sigma_1'}(\mathbf{R}) D^{\frac{1}{2}}_{\sigma_2\sigma_2'}(\mathbf{R}) \tilde{\mathbf{h}}'_{\sigma_1'\sigma_2'}. \tag{4}$$

DeepH-E3 [1] use the complex structure matrix $P = \begin{pmatrix} 0 & 1 \\ -1 & 0 \end{pmatrix}$ to replace representation matrix D by its complex conjugate:

$$\left[D^{\frac{1}{2}}(\mathbf{R})\right]^* = P D^{\frac{1}{2}}(\mathbf{R}) P^\dagger. \tag{5}$$

One can first obtain $\tilde{\mathbf{h}}$ by the tensor product, and then replace the representation matrix with its complex conjugate by the P matrix to construct the final Hamiltonian sub-block $\tilde{\mathbf{h}}P^\dagger$, which has the equivariance described in Eq. (3).

Let us denote the corresponding vector of $\tilde{\mathbf{h}}$ as $\tilde{\mathbf{v}}^{\frac{1}{2}\otimes\frac{1}{2}} = \begin{pmatrix} \tilde{\mathbf{h}}_{\uparrow\uparrow} \\ \tilde{\mathbf{h}}_{\uparrow\downarrow} \\ \tilde{\mathbf{h}}_{\downarrow\uparrow} \\ \tilde{\mathbf{h}}_{\downarrow\downarrow} \end{pmatrix}$, which can be converted to $\mathbf{v}^{\frac{1}{2}\otimes\frac{1}{2}}$:

$$\mathbf{v}^{\frac{1}{2}\otimes\frac{1}{2}} = \begin{pmatrix} 0 & 1 & 0 & 0 \\ -1 & 0 & 0 & 0 \\ 0 & 0 & 0 & 1 \\ 0 & 0 & -1 & 0 \end{pmatrix} \tilde{\mathbf{v}}^{\frac{1}{2}\otimes\frac{1}{2}}. \tag{6}$$

Supplementary Section 2.3. Change of basis

We need to find a change-of-basis that makes the equivariant requirement described in Eq. (2) can be handled in the equivariant neural network (ENN) framework. The basis set of the direct product representation of two spin-1/2 $\{|\uparrow\uparrow\rangle, |\uparrow\downarrow\rangle, |\downarrow\uparrow\rangle, |\downarrow\downarrow\rangle\}$ can be decomposed into the direct sum of spin-0 $\{|0,0\rangle\}$ and spin-1 $\{|1,-1\rangle, |1,0\rangle, |1,1\rangle\}$ representations, and can be further converted to real spherical harmonics of $l=0$ $\{|0\rangle\}$ and $l=1$ $\{|y\rangle, |z\rangle, |x\rangle\}$:

$$\begin{cases} |\uparrow\uparrow\rangle = |1,1\rangle = -\frac{1}{\sqrt{2}}\left(\mathrm{i}|y\rangle + |x\rangle\right), \\ |\uparrow\downarrow\rangle = \frac{1}{\sqrt{2}}\left(|1,0\rangle + |0,0\rangle\right) = \frac{1}{\sqrt{2}}\left(|z\rangle + |0\rangle\right), \\ |\downarrow\uparrow\rangle = \frac{1}{\sqrt{2}}\left(|1,0\rangle - |0,0\rangle\right) = \frac{1}{\sqrt{2}}\left(|z\rangle - |0\rangle\right), \\ |\downarrow\downarrow\rangle = |1,-1\rangle = -\frac{1}{\sqrt{2}}\left(\mathrm{i}|y\rangle - |x\rangle\right). \end{cases} \tag{7}$$

The vector $\mathbf{v}^{0\oplus 1}$ under the basis set of $\{|0\rangle, |y\rangle, |z\rangle, |x\rangle\}$ can be transformed to $\tilde{\mathbf{v}}^{\frac{1}{2}\otimes\frac{1}{2}}$ under $\{|\uparrow\uparrow\rangle, |\uparrow\downarrow\rangle, |\downarrow\uparrow\rangle, |\downarrow\downarrow\rangle\}$ by:

$$\tilde{\mathbf{v}}^{\frac{1}{2}\otimes\frac{1}{2}} = \frac{1}{\sqrt{2}} \begin{pmatrix} 0 & \mathrm{i} & 0 & -1 \\ 1 & 0 & 1 & 0 \\ -1 & 0 & 1 & 0 \\ 0 & \mathrm{i} & 0 & 1 \end{pmatrix} \mathbf{v}^{0\oplus 1}. \tag{8}$$

With Eq. (6), we can obtain the unitary transformation Q, which can convert the equivariant vector under real spherical harmonics to the Hamiltonian matrix sub-block:

$$\mathbf{v}^{\frac{1}{2}\otimes\frac{1}{2}} = Q\mathbf{v}^{0\oplus 1}; Q = \frac{1}{\sqrt{2}}\begin{pmatrix} 1 & 0 & 1 & 0 \\ 0 & -\mathrm{i} & 0 & 1 \\ 0 & \mathrm{i} & 0 & 1 \\ 1 & 0 & -1 & 0 \end{pmatrix}. \tag{9}$$

Supplementary Section 2.4. Realization of equivariance

Under the basis set of real spherical harmonics for the spin degrees of freedom $\{|0\rangle, |y\rangle, |z\rangle, |x\rangle\}$, the equivariant requirement [Eq. (2)] with respect to time reversal becomes

$$\mathbf{v}^{0\oplus 1} = Q^{\dagger}\mathbf{v}^{\frac{1}{2}\otimes\frac{1}{2}} \xrightarrow{\mathcal{T}} Q^{\dagger}\begin{pmatrix} 0 & 0 & 0 & 1 \\ 0 & 0 & -1 & 0 \\ 0 & -1 & 0 & 0 \\ 1 & 0 & 0 & 0 \end{pmatrix} K\mathbf{v}^{\frac{1}{2}\otimes\frac{1}{2}} = \begin{pmatrix} 1 & 0 & 0 & 0 \\ 0 & -1 & 0 & 0 \\ 0 & 0 & -1 & 0 \\ 0 & 0 & 0 & -1 \end{pmatrix} \left(\mathbf{v}^{0\oplus 1}\right)^{*}. \tag{10}$$

We find that under this basis, the equivariant transformation of time reversal becomes simple: the equivariant vectors for the real part of $l = 0$ and the imaginary part of $l = 1$ are time-reversal-even, and the equivariant vectors for the real part of $l = 1$ and the imaginary part of $l = 0$ are time-reversal-odd. Since the equivariant vectors for different l and their real or imaginary part can be constructed separately [1], the requirement [Eq. (10)] can be achieved by adding an extra index marking the "time-revsersal parity" in equivariant vectors.

Supplementary Section 3. Ablation studies on the magnetic moment layer with strict locality

We performed ablation studies to test the performance of the magnetic moment layer with strict locality. We trained two models on datasets of monolayer CrI_3, the model used by xDeepH and the another model treating the atomic and magnetic information on an equal footing. Then we computed the mean squared errors (MSE) of Hamiltonian matrix for test sets. Our experiments indicate that the magnetic moment layer with strict locality inspired by *a priori* knowledge of nearsightedness is more advantageous in both efficiency and accuracy (Supplementary Table 1).

Supplementary Table 1. Results for ablation studies on the magnetic moment layer with strict locality.

	Test set MSE (10^{-6}eV^2)	Number of parameters	Training time for one epoch (second)
Baseline	0.44	2126632	980
No magnetic moment layer	1.19	3013737	1100

Supplementary Section 4. Results of example studies

Supplementary Section 4.1. Monolayer $NiBr_2$

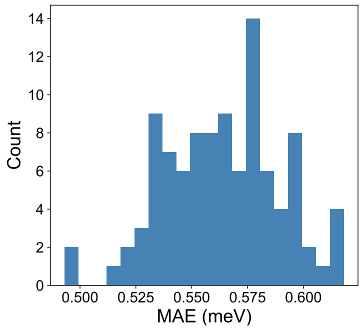

Supplementary Figure 2. Performance of xDeepH on studying monolayer $NiBr_2$. Distribution of the mean absolute error (MAE) of the Hamiltonian matrix element for test sets of monolayer $NiBr_2$. The averaged MAE is 0.56 meV.

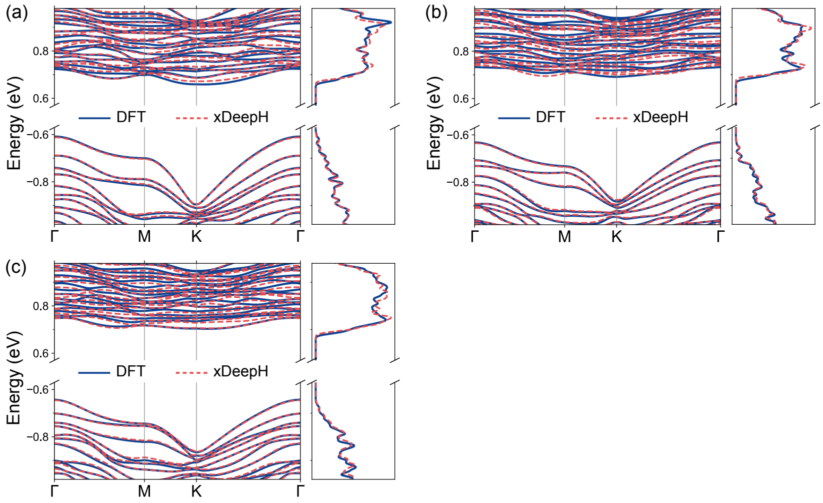

Supplementary Figure 3. Performance of xDeepH on studying band structures and density of states of monolayer $NiBr_2$. Band structures (the left panel) and density of states (the right panel) of the three typical tests samples of monolayer $NiBr_2$ with (a) the smallest, (b) median and (c) biggest MAE of the DFT Hamiltonian matrix element computed by DFT and xDeepH.

The study of $NiBr_2$ (Supplementary Figure 3) indicates that the valence bands can be accurately predicted by xDeepH, whereas the prediction accuracy is obviously lower for the conduction bands. To analyze the discrepancy between valence and conduction bands, we performed an orbital analysis on the band structure of ferromagnetic monolayer $NiBr_2$ (Supplementary Figure 4). The results indicate that the valence and conduction bands are mainly contributed by p-orbitals of Br atoms and d-orbitals of Ni atoms, respectively. Due to the different orbital contribution, electronic states of conduction bands are more sensitive to the change of magnetic configuration. The strongly correlated nature of electronic states would make an accurate prediction of DFT Hamiltonian challenging. This could explain the discrepancy between valence and conduction bands of the system. In contrast, we did not observe similar discrepancy for CrI_3 (see Fig. 3d), although the material also has p-orbital valence bands and d-orbital conduction bands. The underlying reason, however, is unclear, which demands in-depth research.

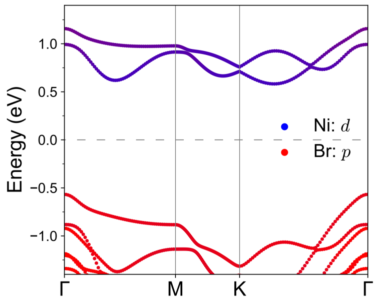

Supplementary Figure 4. Orbital projected band structure of ferromagnetic monolayer $NiBr_2$. Contributions of Ni d-orbital and Br p-orbital are colored blue and red, respectively.

Supplementary Section 4.2. Monolayer CrI_3

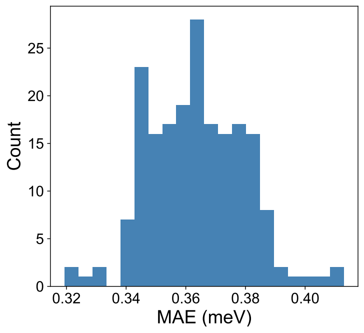

Supplementary Figure 5. Performance of xDeepH on studying monolayer CrI_3. Distribution of the MAE of the Hamiltonian matrix element for test sets of monolayer CrI_3. The averaged MAE is 0.36 meV.

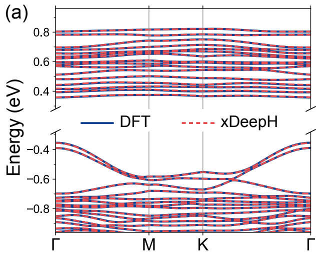

Supplementary Figure 6. Performance of xDeepH on studying band structures of monolayer CrI_3. Band structures of the three typical tests samples of monolayer CrI_3 with the (a) smallest, (b) median and (c) largest MAE of the DFT Hamiltonian matrix element computed by DFT and xDeepH.

Supplementary Section 4.3. CrI_3 nanotubes

The electric susceptibility χ is computed by the formula:

$$\chi^{ab} = \frac{e^2}{\epsilon_0 \hbar} \int \frac{d^3\mathbf{k}}{(2\pi)^3} \sum_{n,m} f_{nm} \frac{r^a_{nm} r^b_{mn}}{\omega_{mn}(\mathbf{k}) - \omega - i\eta}, \tag{11}$$

where a, b and c are cartesian directions, ϵ_0 is the vacuum permittivity, $\hbar$ is the reduced Planck's constant, e is the charge of electron, and η is an infinitesimal broadening factor. $\omega_{mn}(\mathbf{k}) = \frac{E_{m\mathbf{k}} - E_{n\mathbf{k}}}{\hbar}$ and $f_{nm} = f_n(\mathbf{k}) - f_m(\mathbf{k})$ are the difference of band energy eigenvalues and Fermi-Dirac occupations of bands n and m at wave vector $\mathbf{k}$, respectively. r^a_{nm} is the Berry connection when $n \neq m$ or zero when $n = m$. The quantity is calculated by the method developed in Ref. [2].

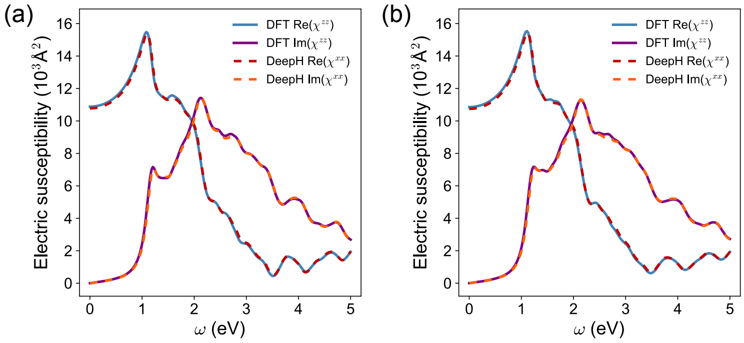

Supplementary Figure 7. Performance of xDeepH on studying the electric susceptibility of CrI_3 nanotube. Real and imaginary parts of electric susceptibility χ^{zz} for (16, 16) CrI_3 nanotube with (a) the non-collinear magnetization normal to the surface or (b) collinear ferromagnetism computed by DFT and xDeepH. The periodic direction of nanotube is defined as the z-axis.

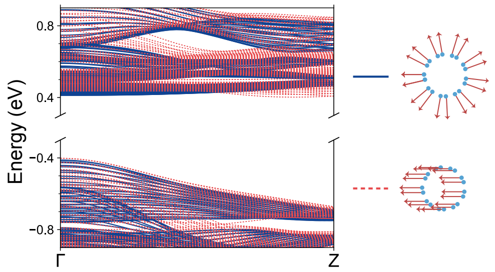

Supplementary Figure 8. Comparison of band structures of CrI_3 nanotube with different magnetic configurations. DFT calculated band structures of (16, 16) CrI_3 nanotube with the non-collinear magnetization normal to the surface (blue) or collinear ferromagnetism (red).

Supplementary Section 4.4. Moiré-twisted bilayer CrI_3

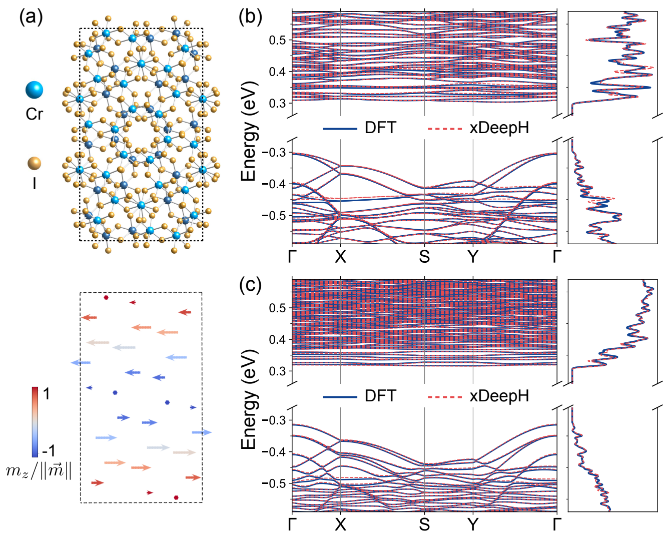

Supplementary Figure 9. Performance of xDeepH on studying 2×1 supercell of twisted bilayer CrI_3 with ferromagnetic or spiral magnetic structures. (a) Schematic atomic structure and spiral magnetic structures of the Moiré-twisted bilayer CrI_3 with twist angle 81.79° including 224 atoms per supercell. Magnetic moments of the top CrI_3 layer are labelled by colored arrows, whose out-of-plane components are shown by the color. The underlying CrI_3 layer is in the ferromagnetic configuration with up magnetic moments. (b,c) Band structures (left) and density of states (right) computed by DFT and xDeepH for the Moiré-twisted bilayer CrI_3 supercell (b) in the ferromagnetic configuration and (c) in the spiral magnetic configuration.

Supplementary Section 5. Computational cost comparison between xDeepH and DFT

To make a quantitative comparison on computational efficiency with DFT, we selected $N \times 1$ supercells ($N = 1, 2, 4, 8 \ldots$) of ferromagnetic monolayer CrI_3 for example studies, computed the DFT Hamiltonian matrices by DFT self-consistent calculations and xDeepH using the same CPU node, and plotted the CPU wall time versus system size in Supplementary Figure 10. The efficiency advantage of xDeepH over DFT is significant and becomes more considerable as the system size increases. Note that xDeepH would work much more efficiently if using GPU nodes and its computational efficiency could be further improved..

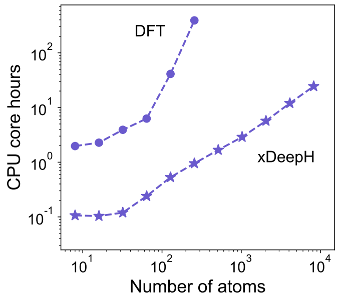

Supplementary Figure 10. Comparison of computation time of DFT and xDeepH. Computation time to construct the DFT Hamiltonian matrices of ferromagnetic monolayer CrI_3 supercells with varying system sizes by DFT self-consistent calculations versus by xDeepH. All the calculations were performed by one compute node equipped with two AMD EPYC 7542 CPUs, although xDeepH would work much more efficiently on GPU nodes.

Supplementary Section 6. Basis set tests

Supplementary Section 6.1. Convergence tests of basis set

For DFT calculations, we have checked the influence of basis set on band structure calculations and confirmed the convergence of basis set before doing routine calculations. Test results on monolayer $NiBr_2$, monolayer and bilayer CrI_3 are shown in Supplementary Figure 11. Further increasing the size of basis set has minor influence on the calculated band structures, implying that the basis set used in our DFT calculations is large enough to ensure convergence.

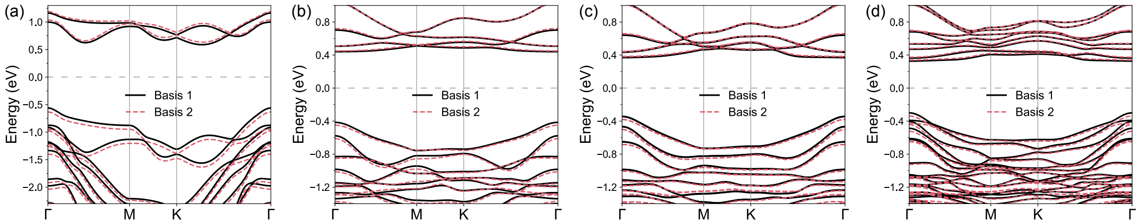

Supplementary Figure 11. Convergence tests of basis set. Band structures of (a) monolayer $NiBr_2$, (b) monolayer CrI_3, (c) bilayer CrI_3 with $C2/m$ interlayer stacking, and (d) bilayer CrI_3 with $R\bar{3}$ interlayer stacking calculated by using different basis sets. The material systems are in the ferromagnetic state for (a,b,d) and in the A-type antiferromagnetic state for (c). "Basis 1" denotes Ni6.0H-$s3p2d1$, Br7.0-$s3p2d2$, Cr6.0-$s3p2d1$, and I7.0-$s3p2d2$ for Ni, Br, Cr, and I, respectively. "Basis 2" denotes Ni6.0H-$s3p2d2f1$, Br7.0-$s3p2d2f1$, Cr6.0-$s3p2d2f1$, and I7.0-$s3p3d2f1$ for Ni, Br, Cr, and I, respectively. "Basis 1" is used in our routine DFT calculations. "Basis 2" is larger in size than "Basis 1".

Supplementary Section 6.2. Training efficiency versus the size of basis set

We also tested the training efficiency of xDeepH versus the size of basis set (Supplementary Table 2). The number of model parameters and time per iteration of training do not increase much for increasing the size of basis set.

Supplementary Table 2. Training efficiency of xDeepH versus the size of basis set. The material system used for test is the ferromagnetic monolayer CrI_3 2×2 supercell with 32 atoms. The neural-network training is performed on an NVIDIA RTX 3090 GPU with a batch size of 1. The basis set used in our routine DFT calculations is highlighted in bold.

Basis functions	Number of parameters	Training time for one iteration (second)
**Cr6.0-$s3p2d1$; I7.0-$s3p2d2$**	**2126632**	**1.6**
Cr6.0-$s3p2d1$; I7.0-$s3p2d2f1$	2530330	1.8
Cr6.0-$s3p2d2f1$; I7.0-$s3p3d2f1$	2903748	1.9

Supplementary Section 7. Comparison between xDeepH and SpookyNet

SpookyNet [3] and xDeepH are developed to provide a complete description of material properties, which not only consider the nuclear degrees of freedom as input, but also take the electronic degrees of freedom into account. Both methods are based on ENN models. It is thus meaningful to compare the two methods. The workflow and application of SpookyNet and xDeepH are illustrated in Supplementary Figure 12. SpookyNet is a deep neural network used to predict the total energy for a given molecular geometry and specified electronic degrees of freedom including the total charge and spin of the molecule. In contrast, xDeepH is a deep neural network used to predict the DFT Hamiltonian for given atomic and magnetic configurations of crystalline materials. Their deep-learning objects and target material systems are distinct. Moreover, the two methods have different applications: The former is applied to study atomic structure, and the latter is used to study electronic structure. In this context, the performance of the two methods cannot be directly and quantitatively compared.

Nevertheless, it is worthwhile to compare the neural network architectures of the two ENNs. SpookyNet takes the total spin of the system into account via a scalar input (a positive integer number). This network does not need to handle rotational and time-reversal equivariance with respect to spin features. Moreover, SpookyNet predicts the total energy, which is invariant under Euclidean symmetry. SpookyNet projects rotationally equivariant features to invariant ones for improving computational efficiency. In contrast, xDeepH includes magnetic features of individual atoms as a vector input, and predicts the DFT Hamiltonian matrix that is more difficult to treat than the scalar output of SpookyNet. All the input, internal, and output feature vectors of xDeepH

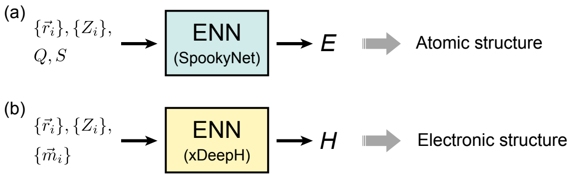

Supplementary Figure 12. Workflow and application of deep-learning models, including SpookyNet and xDeepH, which are used to study (a) atomic structure and (b) electronic structure, respectively. The atomic coordinate ($\{\vec{r}_i\}$) and nuclear charge ($\{Z_i\}$) are inputs to both ENNs. The input of SpookyNet also includes two integer number, the total charge (Q) and spin (S) of the molecule. In contrast, xDeepH considers magnetic moments of individual atoms ($\{\vec{m}_i\}$) as vector input. SpookyNet predicts the total energy (E), and xDeepH predicts the DFT Hamiltonian matrix (H).

are kept equivariant (not simply invariant). Thus, xDeepH can explicitly preserve the rotational and time-reversal equivariance with respect to magnetic features, which is critical for achieving good performance. In brief, the two ENNs have their own advantages: The ENN of SpookyNet is computationally more efficient for predicting invariant physical quantities, and the ENN of xDeepH is more desirable for predicting equivariant physical quantities.

Supplementary Section 8. Summary of the dataset

Supplementary Table 3. Number of parameters of neural network models and the data splitting used for training, validation and test, respectively, in each dataset.

System	Number of parameters	n_{training}	$n_{\mathrm{validation}}$	n_{test}
Monolayer $NiBr_2$	2125259	300	100	100
Monolayer CrI_3	2126632	588	196	196
Bilayer CrI_3	2126632	1498	499	499

Supplementary Section 9. Discussion on applications of xDeepH

Supplementary Section 9.1. Study of magnetic alloys

Magnetic alloys with magnetic moments contributed by impurities can be studied by xDeepH using the following procedures:

1) Prepare DFT datasets: First, a suitable size of supercell is selected, which should be not too small for avoiding artificial interactions between periodic images but not too large for making the ab initio calculations affordable. Then, magnetic impurities are introduced into the supercells with varying impurity concentrations, random impurity distributions, and random magnetic orientations. If the magnetic impurities are prone to be clustered, supercells with clustered impurities should also be carefully considered. The guiding principle is that the generated samples should include diverse kinds of local chemical environments, especially those close to environments in realistic material systems. Finally, DFT calculations are performed to calculate the Hamiltonians of these supercells with random atomic and magnetic structures for the generation of datasets.

2) Train neural network models: Use part of datasets for neural-network training. Test the performance of neural networks by the remaining datasets.

3) Predict electronic properties of new structures: Give a new structure unseen in the DFT datasets. The trained neural networks of xDeepH will be used to predict its DFT Hamiltonian. Then all the electronic properties of this structure in the single-particle picture could be calculated from the predicted DFT Hamiltonian.

As emphasized in the manuscript, xDeepH utilizes the nearsightedness property of electronic matter in order to make predictions on large structures after being trained on small structures, as long as the local chemical environments of the large structures are similar to those of the small structures in the training set. Provided that this principle is followed in the design of the training set, xDeepH could well describe the dependence of ab initio tight-binding Hamiltonian on the local chemical environment, making accurate prediction of electronic structure feasible.

Supplementary Section 9.2. Study of many-body interactions

Theoretically, with the knowledge about the dependence of total energy and effective electronic Hamiltonian (like DFT Hamiltonian) on the atomic structure, one may study material properties related to phonons, electrons, and electron-phonon interactions [4]. Similarly, with the knowledge about the dependence of total energy and DFT Hamiltonian on the magnetic structure, one may investigate material properties related to magnons, electron-magnon interactions and phonon-magnon interactions. Since the dependence of total energy and DFT Hamiltonian on the atomic and magnetic structures can be learned by neural network models, one may use the trained neural network models to very efficiently derive the matrix elements describing electron-magnon coupling, phonon-magnon coupling, etc. In contrast, traditional methods for the calculation of these matrix elements (for instance, ab initio linear response theory) are considerably time consuming. Once the matrix elements are obtained, the Dyson equation is solved to study physical properties, such as the lifetimes of electrons and magnons. The major bottleneck for ab initio linear response theory calculations is to obtain these matrix elements instead of solving the Dyson equation. Intriguingly, the derivative calculations of matrix elements can be efficiently computed by the automatic differentiation technique of neural networks. Therefore, it is highly possible that the xDeepH method will outperform the computationally expensive traditional methods of ab initio linear response theory.

Supplementary Section 9.3. Study of collinear magnetic systems

The xDeepH method is designed to learn non-collinear DFT calculation results of magnetic systems. It is naturally applicable to learn collinear DFT calculation results, considering that the latter corresponds to a special, simplified case of the former. On the other hand, for the non-collinear DFT calculations, one may project the Bloch eigenstates onto the spin-up and spin-down subspaces, and obtain spin-up and spin-down projected band structure or density of states. This is demonstrated by analyzing the non-collinear DFT calculation results of ferromagnetic monolayer CrI3 (Supplementary Figure 13).

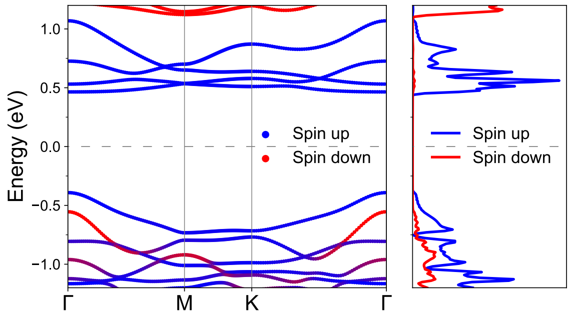

Supplementary Figure 13. Spin-up and spin-down calculations. Spin-up (colored blue) and spin-down (colored red) projected band structure (the left panel) and density of states (the right panel) of ferromagnetic monolayer CrI3 obtained from the non-collinear DFT calculation.

Supplementary Section 9.4. Study of non-periodic systems

It is also valuable from the perspective of deep learning to study spin-dependent electronic properties for non-periodic systems like organometallic magnets. However, xDeepH is originally designed and optimized to study crystalline materials. The neural network models should be re-optimized for the study of molecular systems. The advantage of ENN is obvious in the study of crystalline materials, as demonstrated by xDeepH. For the study of open systems, the benefit of using ENNs becomes less, and more training data would be demanded to achieve comparable prediction accuracy. Moreover, organometallic magnets are physically distinct from solid magnets. The former system usually only has a few magnetic sites that are nearly isolated from each other. Physically, if the magnetic sites are far away from each other, changing their relative spin orientations would essentially not affect the electron density and band structure (or energy levels of molecules). In addition, if the spin-orbit coupling is negligible, the spin and orbital subspaces are decoupled, and then a global rotation of spin configuration would not affect the band structure. Therefore, the change of magnetic orientation typically has minor influence on electronic structure in organometallic magnets. To some extent, the study of organometallic magnets departs from the research of the current work, which could be a new direction for the development of xDeepH.

[1] X. Gong, H. Li, N. Zou, R. Xu, W. Duan, and Y. Xu, General framework for E(3)-equivariant neural network representation of density functional theory Hamiltonian, arXiv:2210.13955.

[2] C. Wang, S. Zhao, X. Guo, X. Ren, B.-L. Gu, Y. Xu, and W. Duan, First-principles calculation of optical responses based on nonorthogonal localized orbitals, New J. Phys. **21**, 093001 (2019).

[3] O. T. Unke, S. Chmiela, M. Gastegger, K. T. Schütt, H. E. Sauceda, and K.-R. Müller, Spookynet: Learning force fields with electronic degrees of freedom and nonlocal effects, Nat. Commun. **12**, 7273 (2021).

[4] G. D. Mahan, *Many-particle physics* (Springer Science, 2000).
